# Local functional descriptors for surface comparison based binding prediction

**DOI:** 10.1186/1471-2105-13-314

**Published:** 2012-11-24

**Authors:** Gregory M Cipriano, George N, Michael Gleicher

**Affiliations:** 1 Department of Computer Sciences, University of Wisconsin-Madison, Madison, WI 53706, USA; 2 Department of Biochemistry, University of Wisconsin-Madison, Madison, WI 53706, USA

**Keywords:** Protein surface shape, Molecular surface, Protein functional surface, Shape descriptors, Protein-ligand docking

## Abstract

**Background:**

Molecular recognition in proteins occurs due to appropriate arrangements of physical, chemical, and geometric properties of an atomic surface. Similar surface regions should create similar binding interfaces. Effective methods for comparing surface regions can be used in identifying similar regions, and to predict interactions without regard to the underlying structural scaffold that creates the surface.

**Results:**

We present a new descriptor for protein functional surfaces and algorithms for using these descriptors to compare protein surface regions to identify ligand binding interfaces. Our approach uses descriptors of local regions of the surface, and assembles collections of matches to compare larger regions. Our approach uses a variety of physical, chemical, and geometric properties, adaptively weighting these properties as appropriate for different regions of the interface. Our approach builds a classifier based on a training corpus of examples of binding sites of the target ligand. The constructed classifiers can be applied to a query protein providing a probability for each position on the protein that the position is part of a binding interface. We demonstrate the effectiveness of the approach on a number of benchmarks, demonstrating performance that is comparable to the state-of-the-art, with an approach with more generality than these prior methods.

**Conclusions:**

Local functional descriptors offer a new method for protein surface comparison that is sufficiently flexible to serve in a variety of applications.

## Background

**Figure 1:**
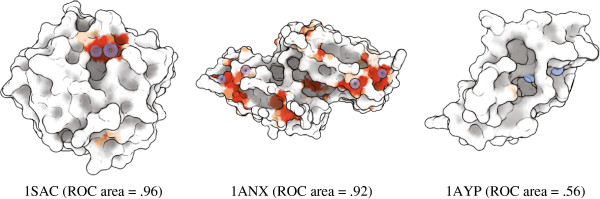
**Shown are the results for three of the calcium ion binding predictions discussed in the results section.** The three examples depict a successful result, a moderate success, and a failure case. In red are areas that the classifier chose as highly likely (>95*%*estimated probability) to bind to calcium. In lighter orange are areas that have between 40% and 95% probability of binding, with the shade of orange indicating approximately where in that range the estimate fell. In white are areas that were deemed unlikely to bind to calcium. The binding locations of the crystal structures are shown as blue spheres with a green point at the center.

**Figure 2:**
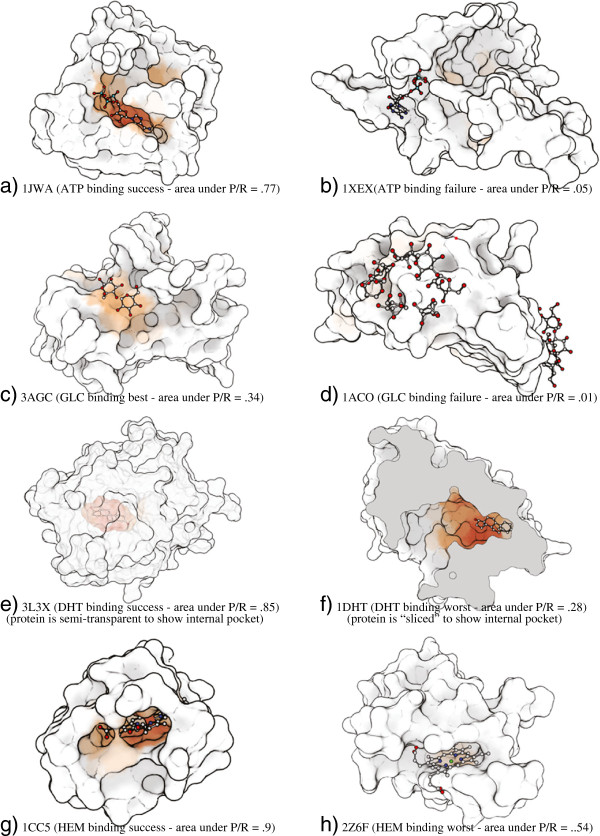
**This image shows representative examples from the multiple-ligand binding test discussed in the results section.** The best and worst examples for each test ligand in the experiment are shown visually. Binding prediciton probability is shown by color on the protein surface: red are areas that the classifier chose as highly likely (>95*%*estimated probability), orange are areas with between 40% and 95% probability of binding.

Molecular recognition in proteins takes place when portions of each partner’s surface are appropriately complementary such that binding can occur. Therefore, the ability to compare regions of surfaces is an invaluable tool for understanding molecular interactions. In this paper, we introduce a novel approach to surface comparison, and show how this approach may be applied to identifying small-molecular binding sites on proteins by comparing the surface of a query protein with those of other proteins known to bind that ligand. Our techniques provide binding prediction probabilities for all positions on the surface of the query protein, as shown in Figures 1 and 2. 

The ability to compare functional surfaces directly is important: while similar phsyio- chemico- interfaces are often created by similar structural scaffolds, we are also very interested in the cases where they are not. For example, in order to predict the function of a protein with little homology to known examples, or compare different mechanisms for similar interactions, comparison mechanisms must be created that operate directly on the physical, chemical, and geometric properties of the interface.

For a specific binding partner, the range of potential interfaces can be quite broad [1]. For example, a ligand may bind in many different ways to different partners. A single descriptor is unlikely to be sufficiently flexible to represent a surface region large enough to encode an entire interface. Therefore, our approach to surface comparison achieves all of these features by representing a protein as a mesh of feature points that sample various physio-chemical properties across the molecular surface. Each sample stores a feature vector that represents a number of properties of a small local region. Larger regions (such as binding interfaces) are compared by combining these smaller regions in a flexible way.

The idea of using a collection of local descriptors has become a standard tool in the Computer Vision community (see [2] for a survey). Small descriptors can be made invariant to irrelevant changes, and combined flexibly to capture larger regions. For images, effective descriptors have been somewhat standardized and evaluated [3], leading to common descriptors such as SIFT [4,5] and SURF [6,7]. A wide variety of approaches for combining these features have been developed, including bag-of-words methods that ignore geometric relationships between features [8-11], flexible template methods that enforce spatial relationships [12-14], and hierarchical methods that combine the advantages of both [15-18].

Recently, others have begun to use the combination of local shape descriptors for protein surface analysis. Notably, Sael and Kihara [19] represent binding sites as collections of points each represented with a Zernike descriptor, and Wallach and Lilien [20] represent binding pockets by representing the shape of subcavities. These methods are most similar to ours, although they both only consider pocket-shaped cavities, and consider only shape, not other physio-chemical properties.

Many different chemical and physical properties affect binding, and any of these properties may effect molecular recognition. Shape^a^ complementarity, electrostatic potential, available hydrogen bond donors and acceptors, hydrophobicity, stereoelectronic effects and other properties *can* determine binding specificity. For any interface, some properties are critical to recognition - but different properties are essential in different interactions [1]. A comprehensive approach to surface comparison must not only consider a range of features, but also must be able to flexibly consider only the appropriate ones in any particular situation. Our approach achieves this by weighting features based on the diversity of examples observed for individual micro-environments.

Beyond shape, electrostatic potential is the most commonly considered physical property (see [21] for a survey of reasons why), and are often considered as part of descriptors. Other properties such as availability of potential hydrogen bond sites are also considered in several methods. Our approach is flexible and able to use any property that can be determined for points on the molecular surface. The method is agnostic to how the properties are computed, allowing standard tools to be used in creating the information encoded in the descriptors and compared. A key element in our approach is that these properties are used independently: the structure of the protein is used to compute the surface properties, however once these properties are computed, the internal structure of the protein is not considered by our methods.

### Properties of descriptor-based methods

Surface comparison is a valuable tool for a number of uses including docking (both protein-protein and protein-ligand), function prediction (annotation), prediction of interactions, classification, and detailed mechanistic analysis of known interactions. In order to create a comparison technique that may serve all of these applications, we sought to create a surface descriptor and comparison technique that has the following properties which, to our knowledge, no single prior method completely satisfy. We note at the outset that we have only incorporated our comparison approach and descriptor in a limited range of applications, and have not yet demonstrated the advantages of all of these features in our method.

#### Capable of matching despite flexibility

The range of interfaces that might serve for an interaction is both specific, yet flexible. Many different interfaces may serve to recognize a partner, and a particular molecule may move so that the interface it shows in an example structure may not be identical to the one it presents in a complex with a partner. The key challenge of comparison-based approaches is to be able to find functionally equivalent interfaces despite these flexibilities. Techniques attempt to address this challenge using a variety of strategies that fall into two broad categories: creating complex descriptors that are flexible and (therefore, hopefully) invariant to these differences, or describing interfaces as collections of simpler, invariant elements and incorporating flexibility into the matching process. Our work falls into the latter category as we believe this approach is more likely to provide the advantages we seek.

#### Not based on sequence

There are many ways that a protein can “implement” the required interface for a particular interaction. Analagous interfaces often result from homologous sequences, so that sequence-based matching methods are often successful. There is a vast literature of sequence-based tools. However the search for functionally equivalent but less related proteins is important and under utilized. Our work is a member of a growing category of structural bioinformatics work that does not consider sequence.

#### Not based on residue positions and sets

Because it is ultimately the physical and chemical properties of the interface that drive recognition, our approach considers these properties directly, not the structural features that cause them. In contrast, many structual bioinformatics techniques consider the residues that make the interface, abstracting interfaces as sets of residues (e.g. [22-24]) or characterizing them as the position of the *Cα*carbons of the residues. Such approaches may be problematic as they do not consider the details of residue configurations, and may not account for different configurations of residues that achieve analogous interfaces. Some methods (e.g. [25]) use equivalence rules or tables to combat the latter problem, by noting which residues often serve similar functions.

#### Not based on a single large descriptor

Binding interfaces are sufficiently complex and diverse so sufficient detail must be encoded. Creating a single descriptor for an entire interface requires developing a descriptor that can capture this range, and be invariant to the various forms of diversity that equivalent interfaces can have. While there are numerous attempts to create descriptors with such flexibility (such as [1,19,23,26-29]), we instead choose a different strategy: describing interfaces as collections of more local descriptors.

#### Not only for pocket shaped regions

While most small molecule interactions involve pocket-shaped voids [30], methods limited to comparing such pockets are inappropriate for protein-protein interactions, for ligands that “stick” to the exterior of proteins, or for the relatively common case where the binding pocket is much larger than the actual binding interface [29]. Many prior shape descriptors used for surface comparison, including moments [31], distance profiles [23,26], and Spherical Harmonics [29,32], can only represent globular (roughly spherical) shapes or enclosed volumes. [27] consider many properties of cavities in determining if they are likely binding sites.

#### Not requiring pre-identified pockets

While geometric pockets can often be reliably identified, a method restricted to comparing identified pockets is limited to pocket interfaces, and subject to issues in pocket localization [29]. Many methods include pocket identification as their first step, such as [19,20,22,23,29].

#### Not requiring precise determination of dense or critical points

Approaches using surface representations may use a dense sampling of the surface [33,34], or sparser point sets. Dense samples can be problematic as they capture very local information and therefore require large groupings to be considered, which becomes challenging if the surfaces to be compared are not sampled equivalently. They also rely on the sampling density being aligned with the scale of the features to be matched. Sparse points may be selected by geometric criteria [35-38] or as chemically significant points [39-42]. Sparse points afford more efficient and flexible matching, although [34] dismiss sparse points because the reduced information reduces the sensitivity and selectivity of the methods, and makes them more sensitive to changes in the points. Our work provides the benefit of both: we avoid the down sides of sparse points by using neighborhood descriptors and flexible matching.

#### Capable of considering interfaces of varying size and shape

Many methods, particularly approaches that create single large descriptors for entire interfaces, are limited in the range of interfaces they can match. For example, the various variants of Feature (c.f. [43-45]) describe spherical regions, and therefore has primarily been used to find interfaces for spherical partners (such as metal and calcium ions). S-Blest [46] similarly characterizes a spherical region around amino acids. Similar methods have mainly served to recognize small ligands (e.g. [47] for glucose molecules). Our approach can scale to a range of interface sizes and shapes.

#### Capable of combining a range of physico-chemical features

Our approach can create rich descriptions of many different chemical and physical properties across an interface. While many descriptor-based approaches do consider multiple properties, most of the ones that handle shape in a detailed and flexible way do not. A range of local shape descriptors have been developed by the geometric modeling community for applications including shape matching, such as [48-52]. While some of these have been adapted for use in molecular applications (e.g. [28]), none consider properties in addition to shape.

#### Capable of selecting from many features

In combining many types of features leads to large feature sets. In order to achieve good performance, methods must perform some form of feature selection (or use a machine learning technique that performs feature selection implicitly). For example, [27,47] both use random forest techniques on a large collection of features that describe entire binding pockets. Our method weights features for different parts of the interface, rather than doing a single weighting for the entire interface.

## Methods

The key idea of our method is to describe the functional surface of a protein as a set of descriptors, each positioned at a point on the solvent accessible surface. For each surface sample, we compute a descriptor vector that describes the local shape and physio-chemico-properties. These descriptors can be precomputed for proteins of interest.

While our descriptors and approach may be applicable to a number of problems, we focus on a single one for our initial validation study: identifying potential ligand-specific binding sites. Specifically, for a particular target ligand we create a classifier that finds locations on the surface of a query protein that are similar enough to be likely to bind the ligand. The classifier is constructed by using a training set of example compounds containing bound ligands that share moeities with the target ligand. Once constructed, the classifier can be used on the surface of a query protein, providing a probability for each point on the query’s surface that the point is part of an interface with the target ligand.

Our approach is comprised of three phases, which are overviewed here and detailed in sections below:

### Descriptor computation

For each protein used in our approach (either as part of the training set or a query), we compute a set of functional descriptors. That is, for a uniform collection of points on the solvent accessible surface, we compute a vector of physical and chemical properties. While the structural information of the protein (e.g. the PDB file) is used to compute the descriptors, this internal structure of the protein is not used in any other phase.

### Classifier construction

For a given target ligand, we construct a classifier comprising a set of per-atom classifiers for each atom in the ligand, and inter-atom distance information. Because we may not be able to find a sufficient number of example complexes containing the ligand, our approach breaks the target ligand into smaller moeities. For each moeity, our approach creates a training set of complexes with a bound ligand containing the moeity. For each atom in the moeity, the training set is used to create a classifier for surface descriptors likely to be near the atom when bound. The statistics of the distances between moeity atoms in the examples are also computed.

### Classification

For a given query protein, the classification procedure gives a field over the surface that measures the likelihood that the target ligand may bind at each location. Classification operates in a fine-to-coarse fashion. First, each per-atom classifier is applied to the protein surface giving a probability field for each one. Second, these probability fields are combined to find areas where the correct combination of atoms are likely to bind at an appropriate distance from each other, giving a prediction of where the moeities are likely to bind. Finally, the moeity predictions are combined to give the probabilities for target ligand.

### Descriptor computation

Our method begins by computing the solvent accessible surface as a mesh. Each vertex of the mesh serves as a surface sample, and the connectivity of the mesh will be used to approximate distances across the surface. While any method could be used, our experiments use the MSMS program [53] to create the meshes, and the corner cutting approximate geodesic method of [54] to compute distances between points.

As a convenient solution, we use the vertices formed during tessellation with MSMS [53], with the sampling density set at approximately 3 points per Å^2^. This means, for instance, that a carbon atom, having a radius of 1.74 Å and thus a surface area of about about 10.7 Å, will contain around 30 samples. By visual inspection, this appears to be enough to represent all geometric detail at the smallest scale.

For each surface sample, we compute a *descriptor vector* that encodes the local shape and physical and chemical properties. Shape is encoded using the multi-scale shape descriptors of [54], that provide larger-scale analogs of curvature, anisotropy, and curvature variability of the surface neighborhood around the sample point. Electrostatic potential is computed as a volumetric property (our implementation uses the APBS program [55]), and sampled at the surface points. For the experiments described in this paper, APBS is used with its default parameters and a 1Å resolution. While there is evidence that detailed physical computations are not essential for the kinds of applications our approach targets [56], we have chosen to use standard methods in our initial experiments. Hydropathy is also computed and sampled at each sample point using the method of [57].

**Figure 3:**
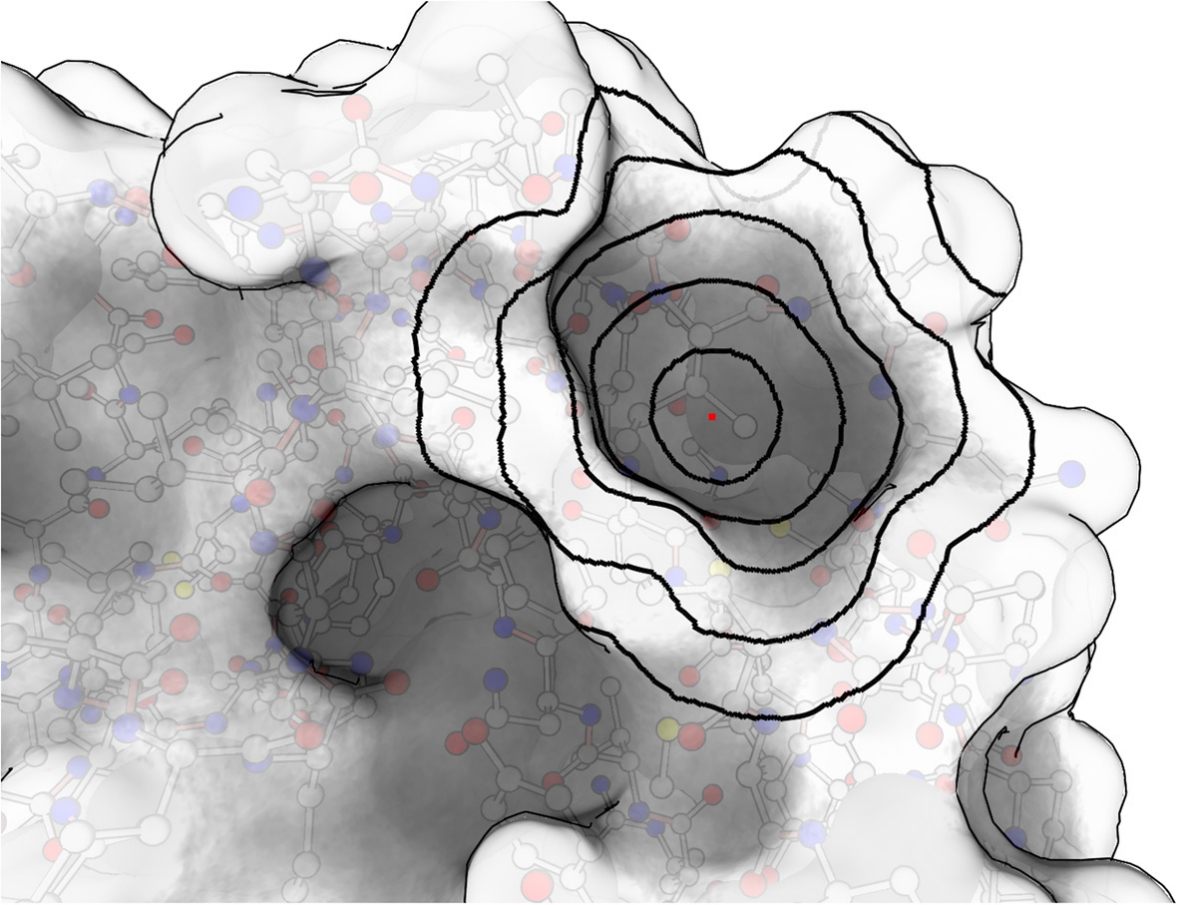
Shown here are, for one sample point, the disc-shaped patches of each radii used in the functional surface descriptor: 1.6Å, 3.2Å, 4.8Å, 6.4Å and 8Å.

Each of the sampled, spatially varying properties (curvature, anisotropy, curvature variance, hydropathy, and electrostatic charge) are sampled at several different scales. A scale is specified as a geodesic disc around the sample point. For example, electrostatic charge at the 8Å scale is the weighted average of all sample points within 8Å geodesic distance of the sample point. Each property is sampled at 5 scales: 1.6Å, 3.2Å, 4.8Å, 6.4Å and 8Å. These also map to the scale of important biological features, the first to the size of an atom, the next two to the size of a residue, and the last two to the size of small pockets (see Figure 3 for a depiction of these sizes).

The set of scales was chosen to balance the compactness of the feature vector with the need to encode as much information as possible. This set was determined empirically by evaluating the covariance of curvature and electrostatics over 20 scales across a corpus of molecules. The smallest scale, 1.6Å, was chosen to be small enough to account for atomic features, but large enough that every patch of this size contained at least five vertices (not counting the center point): the minimum number to avoid an under-constrained fit. The largest size, 8Å was empirically chosen to capture pockets of approximately the size of the largest moiety to be characterized. Remaining scales were removed if their statistical correlation with any other scale approached 1.0, which indicated that they conveyed little new information.

An additional set of features are used to encode the relationship of each sample point to certain chemical features. Each descriptor vector includes the Euclidean distance to the nearest of each of the following: non-polar backbone atom, aromatic sidechain atom, nitrogen backbone atom, aliphatic sidechain atom, oxygen backbone atom, sulphur sidechain atom, amide nitrogen sidechain atom, amide oxygen sidechain atom, typtophan sidechain atom, hydroxyl sidechain atom, charged oxygen sidechain atom, charged nitrogen sidechain atom, potential external hydrogen bond donor, and potential hydrogen bond acceptor site. A final feature records the amount of the “outside” world visible from the sample point (as used for ambient occlusion lighting [58]). This feature is akin to that used by [59].

Because each feature spans a different range of values, samples must be normalized so that the scale of the distributions for each feature are similar. Normalizing each protein independently leads to a per-protein bias which made comparison between proteins inconsistent (as features may have quite different statistics from one protein to the next). Therefore, we normalize feature distributions by computing a mean and standard deviation over a set of proteins randomly selected from the entire PDB (100 in all). Each feature, then, is normalized against these values before being used.

The result is that for each vertex of the mesh, we have a vector of length 40. A detailed summary of this feature vector is provided as Table 1. All of these properties in the feature vector are invariant to position and orientation. It is straightforward to add additional properties to the feature vector, provided they can be computed for each surface sample. For example, we might add properties such as travel distance [60,61]. 

**Table 1:** A list of each feature contained within our surface descriptor

**Feature #**	**Name**	**Description**
1	% Visibility	Percentage of outside world visible from point
2	Non-Polar Backbone	Distance to the nearest Non-Polar Backbone Atom
3	Arom. Sidechain	Distance to the nearest Aromatic Sidechain Atom
4	Aliph. Sidechain	Distance to the nearest Aliphatic Sidechain Atom
5	N Backbone	Distance to the nearest Nitrogen Backbone Atom
6	O Backbone	Distance to the nearest Oxygen Backbone Atom
7	S Backbone	Distance to the nearest Sulpher Sidechain Atom
8	Amide N Sidechain	Distance to the nearest Amide Nitrogen Sidechain Atom
9	Amide O Sidechain	Distance to the nearest Amide Oxygen Sidechain Atom
10	Trp Sidechain	Distance to the nearest Trypophan Sidechain Atom
11	Hydroxyl Sidechain	Distance to the nearest Hydroxyl Sidechain Atom
12	Charged O Sidechain	Distance to the nearest Charged Oxygen Sidechain Atom
13	Charged N Sidechain	Distance to the nearest Charged Nitrogen Sidechain Atom
14	Anisotropy (1.6 Å)	Patch anisotropy, with radius: 1.6 Å
15	Anisotropy (3.2 Å)	Patch anisotropy, with radius: 3.2 Å
16	Anisotropy (4.8 Å)	Patch anisotropy, with radius: 4.8 Å
17	Anisotropy (6.4 Å)	Patch anisotropy, with radius: 6.4 Å
18	Anisotropy (8 Å)	Patch anisotropy, with radius: 8 Å
19	Curvature (1.6 Å)	Patch curvature, with radius: 1.6 Å
20	Curvature (3.2 Å)	Patch curvature, with radius: 3.2 Å
21	Curvature (4.8 Å)	Patch curvature, with radius: 4.8 Å
22	Curvature (6.4 Å)	Patch curvature, with radius: 6.4 Å
23	Curvature (8 Å)	Patch curvature, with radius: 8 Å
24	Curvature Var. (1.6 Å)	Variance of curvature within patch of radius: 1.6 Å
25	Curvature Var. (3.2 Å)	Variance of curvature within patch of radius: 3.2 Å
26	Curvature Var. (4.8 Å)	Variance of curvature within patch of radius: 4.8 Å
27	Curvature Var. (6.4 Å)	Variance of curvature within patch of radius: 6.4 Å
28	Curvature Var. (8 Å)	Variance of curvature within patch of radius: 8 Å
29	Hydropathy (1.6 Å)	Weighted avg. hydropathy over patch of radius: 1.6 Å
30	Hydropathy (3.2 Å)	Weighted avg. hydropathy over patch of radius: 3.2 Å
31	Hydropathy (4.8 Å)	Weighted avg. hydropathy over patch of radius: 4.8 Å
32	Hydropathy (6.4 Å)	Weighted avg. hydropathy over patch of radius: 6.4 Å
33	Hydropathy (8 Å)	Weighted avg. hydropathy over patch of radius: 8 Å
34	Charge (1.6 Å)	Weighted avg. charge over patch of radius: 1.6 Å
35	Charge (3.2 Å)	Weighted avg. charge over patch of radius: 3.2 Å
36	Charge (4.8 Å)	Weighted avg. charge over patch of radius: 4.8 Å
37	Charge (6.4 Å)	Weighted avg. charge over patch of radius: 6.4 Å
38	Charge (8 Å)	Weighted avg. charge over patch of radius: 8 Å
39	Hyd. Bond Donor	Distance to nearest potential external hydrogen bond donor
40	Hyd. Bond Acceptor	Distance to nearest potential external hydrogen bond acceptor

Note that features 14 - 38 are weighted according to distance (from center vertex) and point area (i.e. the sum of the areas of all adjacent triangles).

We note that by sampling the molecular surface densely, for each vertex of the detailed solvent accessible surface mesh, we have over-sampled the surface and have considerable redundancy in the data. Grouping similar samples is important for efficiency: however, at this point, we do not know which of the properties are important. The distance metric for grouping must be based on an understanding of the sensitivity of a particular application, so grouping is deferred until such sensitivities can be determined.

We also note that once the various properties of the protein are computed, the actual structure of the protein is not considered in any of the subseqent analyses. Only the mesh and its associated descriptors are used.

### Classifier construction

For a given target ligand, a classifier is constructed that can be applied to query proteins. Construction has three main steps: first, a set of training examples is drawn from the Protein Data Bank (PDB) for each moeity of the ligand; second, these training examples are used to create per-atom classifiers for each atom in the ligand; and third, inter-atom distances are computed.

This learning process is broken down to the level of the atoms themselves, rather than the entire ligand. We base this decision on two fundamental assumptions, which are drawn from experimental observations. First, we assume that *the preferred microenvironment for each atom in a ligand is different*. This difference may be small, as in the case of two neighboring atoms of similar size and polarity, or large, as in, for instance, the difference between the predominantly negatively-charged phosphate chain in ATP and the positive adenine moiety. Intuitively, this assumption is justified by the lock-and-key principle: each atom in a moiety has a particular property (polarity, shape, etc.) which is different from all other atoms in the moiety. Thus complementarity implies that the matching surface for each of these atoms will also have different, complementary properties.

The second assumption is that, for a given atom, *the microenvironment surrounding that atom is consistent across all proteins to which it binds*. For this to be true, in the samples in close proximity to a given binding atom, the feature vectors for each sample must form one or more clusters in feature space (i.e. its ‘signature’). This implies that samples binding a given atom may be distinguished from samples that don’t, given a classifier trained on a corpus large enough to encompass all of the possible binding microenvironments of an atom. This assumption is key to the success of our work: if this were not true, then our binding prediction algorithm would be impossible, as separating positive and negative examples would itself be impossible.

The flexibility in our approach comes not only from accounting for the range of micro-environments in which a particular atom may bind, but also by allowing for relative movement and re-arrangement between atoms.

#### Selecting training examples

For each atom in the ligand, we build a classifier that can identify its preferred micro-environment. To create a classifier that is sufficiently general, we need sufficient examples that capture the diversity of binding interfaces for the particular atom in the ligand. Unfortunately, for many ligands, the Protein Data Bank does not have a diverse enough set of example complexes. Therefore, rather than simply using examples of the ligand, our method breaks the ligand into smaller moeties, and builds a training corpus for examples containing this moeity. For example, rather than building a classifier for a particular carbon of ATP, our method builds a classifier for the C1 carbon of the adenine moeity that is part of ATP. This allows building the classifier from a training set of all proteins binding all ligands that contain the adenine moeity, rather than the smaller potential training set of those proteins binding ATP. Moeities are manually identified when the target ligand is specified.

For each moeity in the target ligand, we select a training corpus from the PDB using Algorithm 1. In order to avoid redundancy, the algorithm selects at most one example from each cluster of homologous proteins. We use the clusterings produced by BLASTCLUST (using data available at ftp://resources.rcsb.org/sequence/clusters/). Proteins are considered homologous if they contain greater than 95% sequence identity. For our experiments, this leaves 27551 clusters (out of 66961 proteins).

##### Algorithm 1. The corpus-building algorithm

Descriptors for all proteins used as examples are computed as discussed above.

#### The training algorithm

In this step, a classifier is trained for each atom in a moiety. At a high level, the algorithm proceeds as follows: first, for every atom in the moiety to be trained, for each example protein surface in the training set, all samples are found that come within 1.6 Å of the atom. These are classified as positive examples. Negative samples are randomly chosen from the remainder of the surface. Though there are many more negative samples on a surface than positive, adding more negative examples does not necessarily improve predictive accuracy [62]. Further, classification time increases with the number of samples. The negative set is therefore chosen to be the same size as the positive set. Both sets are then used to train a classifier to recognize that atom. This process is repeated for every atom in the moiety.

Given the positive and negative sets, we train a SVM classifier with a radial basis kernel [63,64]. We used the Weka program [65] for creating the classifiers. Working with the Weka framework allowed us to try other classifiers, however the radial basis SVMs were found to have the best combination of classification speed and accuracy.

**Figure 4:**
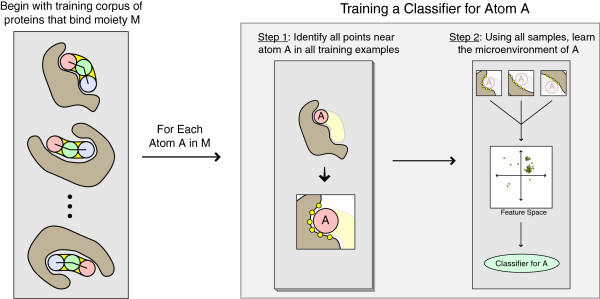
A visual depiction of Algorithm 2, for training a classifier to recognize the environment surrounding a specific atom, given a corpus of examples of that atom’s binding.

Refer to Algorithm 2 for a detailed description of this process, and to Figure 4 for a visual depiction.

##### Algorithm 2. The algorithm for training a set of classifiers ***C***_***A***_ on a specific moiety ***M*** given a test corpus containing ***M***

#### Atomic distance measurement

As discussed below, our algorithm uses structural knowledge about the moiety as a guide for combining atomic surface predictions. Specifically, to combine a prediction for atom A with a prediction for atom B, it needs to know the allowable range of distances that A and B can be from one another with respect to the moiety that contains them both; it would make no sense to combine predicted locations for both atoms that were either too close or too far from one another to be physically plausible.

Thus the final aspect of training for a moiety, separate from building the per-atom classifiers themselves, is the computation of all-pair distances between atoms in that moiety. Our algorithm does this by finding the minimum and maximum bound for the distance between each pair of atoms in all ligands encountered during training.

**Figure 5:**
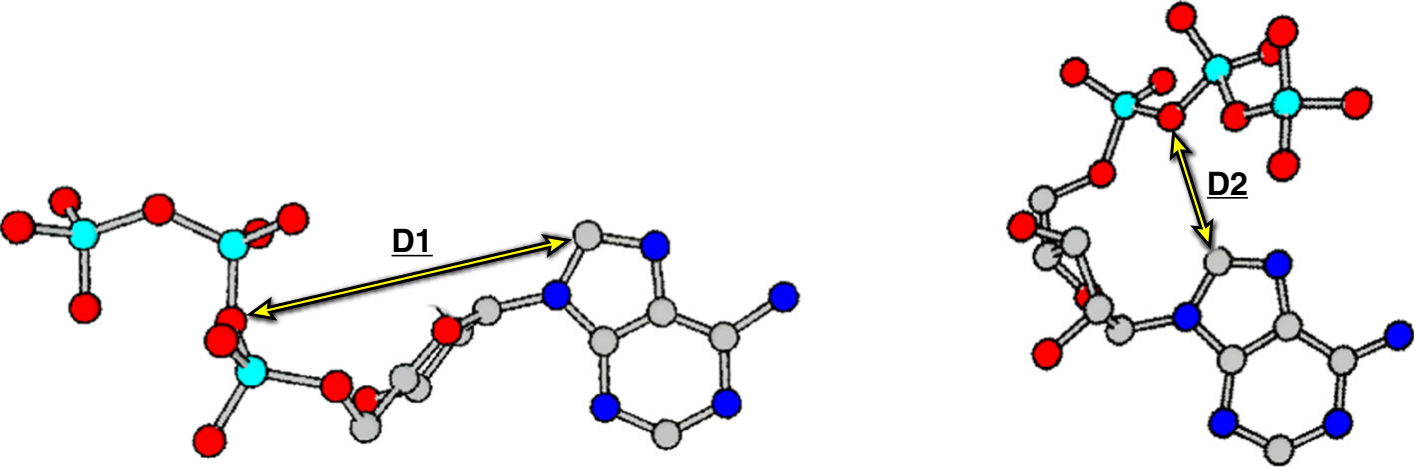
**Shown here are two possible conformations for Adenosine Triphosphate (ATP).** Note that the distances between atoms within the rigid adenine moiety do not change. Distances between non-rigid components, such as those between the ‘C8’ and ‘O3A’ atoms, may change dramatically. As these will be used later to combine atomic predictions, the observed minimum and maximum distances between each pair of atoms are stored during the training phase.

For rigid structures, the bounds for these distances will be quite tight, as any two atoms will appear at similar distances from one another across the corpus. But for flexible moieties, this process accounts for their structural flexibility (see Figure 5).

The computational time taken in this step is negligible.

### Performing classification

**Figure 6:**
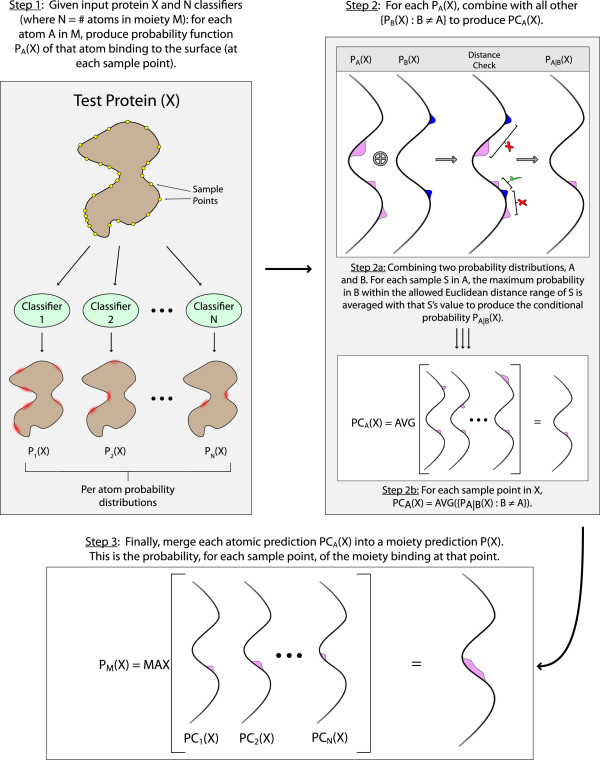
Prediction Phase: combining atom surface functions to predict a ligand.

To predict where a target ligand might bind a query protein surface, the per-atom classifiers created in the previous section are combined. The process works in a fine-to-coarse fashion: For each sample of the query surface, each of the atom classifiers are applied to determine the potential binding locations for each atom in the moeity. These predictions are then combined to find places where the atoms of the moeity may bind in an appropriate relative position. Refer to Algorithm 3 for a description and Figure 6 for a visual depiction of this process. The approach is detailed in the subsections below.

#### Algorithm 3. Overview of the moiety-prediction process: using trained classifiers to predict the presence and location of a moiety on the surface of a molecule

#### Reducing sample count

As was noted above, a full set of samples is used in the training phase. This is done because during training, nothing is yet known about the statistics of the surface features, so there is not yet enough information to determine which features are important for discrimination and which are not. Therefore, the learning algorithm cannot identify ‘redundant’ points — those which are both physically adjacent on the surface and also close in feature space along the features which matter. This redundancy slows the training process, as more points must be considered.

**Figure 7:**
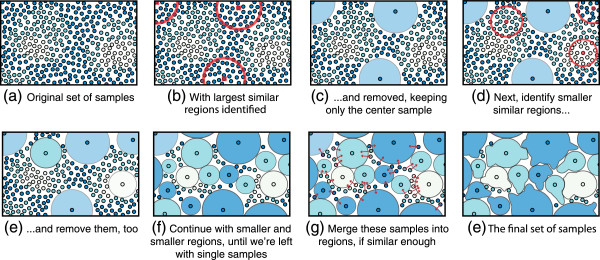
**An illustration (in 2D) of how our method for grouping samples on the 3D surface works.** In this illustration, each circle represents a sample; samples having similar values in feature space are given the same color. The algorithm proceeds as follows: starting with a radius *R*, identify discs of radius *R* that have minimum average distance (in feature space) between elements in the disc. Replace the best non-overlapping discs with the sample in the center of each disc. Repeat, each time reducing the size of the disc. When complete, there will still be samples not contained in a disc. Merge those into neighboring discs if their distance from the center sample is less than a threshold *T*. The resulting center samples are used for surface prediction. In all results, *R*= 4Å and *T*=.25.

To improve the speed of moeity prediction, we reduce the number of sample points considered by grouping them into clusters. We have developed a fast sample-grouping algorithm to perform this reduction, which is described in Algorithm 4, and depicted in Figure 7.

##### Algorithm 4. A greedy algorithm for grouping samples on the surface according to feature distance

The algorithm is designed to repeatedly find the largest possible disc (less than 4 Å in radius) such that all samples in this disc have an average feature distance less than some threshold *T* (in this case.5, or equivalent to half of the standard deviation over the corpus) from one another. When no more discs of a particular size can be found, the search begins again with a smaller disc, and repeats down to a radius of.5 Å. Finally, since some samples were not assigned, those are merged into any neighboring discs if the feature distance between that sample and the sample in the center of the disc is less than *T*.

Note that because each atom in a moiety has a slightly different microenvironment, each atom has a different set of ‘important’ features. We define important features to be those that, over the training set, have less variance than background, which, because each feature is normalized, equals 1. It is only these features that are used in the distance computation. Thus, this algorithm is run for every atom, and every atom gets its own set of non-redundant samples.

By grouping similar samples and culling all but one representative sample for each group, this algorithm significantly reduces the number of samples on the surface, usually anywhere from 5x to 20x, depending on overall sample similarity. This has a significant impact on prediction speed, as described below. Note that grouping samples in this way could result in an overestimation of the distance between samples. Our algorithm compensates for this effect during each range search by relaxing the inter-atom distance constraints by the radius of the patch.

#### Predicting for an atom

In our algorithm, predicting the location of a moiety begins with predicting the location of each atom in that moiety. In this phase, each atom classifier is run over the non-redundant samples produced for that atom on the test surface. For each sample, the classifier produces a probability that the atom on which it was trained binds to that sample.

For a surface *X*, the end result is a function over the samples on *X*, *P*_*A*_(*X*), indicating the likelihood that an atom *A* would bind at each sample. This is Step 1 in Figure 6.

#### Predicting moeties:combining atom predictions

Once a set of probability functions *P*_*A*_(*X*) have been computed over the set of samples, with one function for each atom *A* in moiety *M*, the next step is to combine these predictions into a single function predicting the likelihood that *M* binds at each sample, which will be called *P*_*M*_(*X*). Note that since the culled set of samples is different for each atom, *P*_*M*_(*X*) will be over the *complete* (i.e. non-culled) set of samples.

As shown in Step 2 of Figure 6, as an intermediate step toward this goal, the algorithm first computes for each atom *A*, *PC*_*A*_(*X*) : the probability that *A* binds to each sample *S* in *X*, given the probabilities computed for all other atoms in *M*. In this step, the distance information that was computed above is used when combining the probability functions for two atoms.

To recap: in a given ligand, any two atoms *A* and *B* only appear within a specific range of distances from one another. Therefore, if *A* has a high probability of binding to surface *X* at a point *S*, then we can *further confirm the legitimacy* of that prediction by seeing if there is a similarly high probability of *B* binding to *X* within the correct distance. Thus, *PC*_*A*_(*X*) refers to the probability of *A* binding at each sample *confirmed by all other probability functions*. This is described in detail in Algorithm 5.

##### Algorithm 5. Algorithm for combining a set of probability functions ***P***_***A***_(***X***), which indicate the likelihood of an atom ***A*** binding at samples on surface ***X***, into set of probability functions ***PC***_***A***_(***X***), which indicate the likelihood of ***A*** binding at each sample ***confirmed by all other probability functions***

Note that in merging individual probabilities *P*_*A*_(*X*) into conditional probabilities *PC*_*A*_(*X*), neighboring probability functions are averaged within the allowed distance window. This was chosen instead of using the ‘maximum’ neighboring probability so that spurious high probability scores do not have undue influence on the final conditional result.

Finally, as shown in Step 3 in Figure 6, all *PC*_*A*_(*X*) are merged into a final *P*_*M*_(*X*) by taking the maximum probability over all *PC*_*A*_(*X*). The intuition here is that false positives have already been accounted for by the previous step, so each *PC*_*A*_(*X*) should contain a good prediction of the binding location of *A* within moiety *M*. Therefore, merging these predictions will produce a final prediction of the locations where *M* will bind.

### Predicting ligands: merging moiety predictions

For those ligands that have been broken into multiple moieties, we now have multiple predictions *P*_*M*_(*X*) over the surface for the locations of moieties. In many proteins, only a subset of the moieties may bind to the surface of the protein, with the rest floating off the surface. Further that subset is not consistent: ATP, for instance, has three moieties, and within its training corpus, all possible combinations of these three moieties are bound to the surface.

The prediction that any one moiety binds, therefore, may be enough to predict ligand binding. We adopt a heuristic for merging moieties that simply returns back the highest probability over all moiety predictions as the final probability. In other words, for each sample *S*, *P*(*X*)[*S*]= max_*M*∈*L*_*P*_*M*_(*X*)[*S*]. This method is robust against partial matches, as described above, but as a consequence is likely to confuse ligands that share many similar moieties (ADP and ATP, for instance). We consider this limitation in the discussion.

## Results and discussion

### Results

In this section, we describe a series of experiments designed to show the viability of our approach. All experiments involve predicting binding sites given a query protein and a target ligand for which the method has been trained. Our method considers the entire surface of the query protein: it identifies regions on which there is a high enough probability that they may be a binding site. After the surface mesh and descriptors are computed, our method does not consider the atomic coordinates inside the mesh: it relies solely on the geometric and physical properties encoded by the descriptor mesh.

The results of our methods are a probability for each surface point on the query protein. These results are most effectively used by visual inspection, to identify interesting regions of the query molecule, such as shown in Figures 1 and 2. However, in order to validate the quality of these results, we have performed a series of experiments that provide quantitative results for benchmark problems.

To evaluate predictive performance quantitatively, our tests consider proteins with known binding pockets where the PDB structure includes the ligand. Quantitative assessment of performance requires determining the percentage of the surface points correctly identified as being part of the binding interface. As our method produces a probability, such a binary grading requires the choice of a threshold to balance false negatives and false positives. Rather than selecting arbitrary values, we assess performance by analyzing precision-recall and receiver operating characteristic (ROC) curves.

Quantitative assessment also requires a definition of which points are considered as part of the interface. We consider a point to be part of an interface (e.g. a true positive) if it is within 1.6Å of the surface of any atom in the ligand of interest which binds to that surface. This probably under-states our results, as the actual interface is most likely larger in area because the crystal structure only shows one possible position of the ligand. A successful result not only requires that the trained ligand binds the query protein, but that the interface region is correctly localized.

As discussed in the Background, there are many successful approaches for protein-ligand binding prediction. Our approach is different in that it attempts not only to predict that the query protein will bind the ligand of interest, but also to determine the surface region where this interaction is likely to occur. Also, we reiterate that most of the prior successful approaches have restrictions (described in the Background) that our approach does not. For example, our approach does not require topologically spherical pockets. For these reasons, it is difficult to compare our approach with the prior art. The previous methods are effectively specialized to the problems they address, and we would expect them to provide better performance than an approach such as ours that does not have their restrictions and uses more limited information.

In this section, we describe a series of experiments where we apply our approach to the problems addressed by prior methods. We have chosen problems for which prior approaches to binding prediction perform well, so that we know the test cases are tractable. While we would not expect our method to be competitive with more specialized approaches, our goal is to show that it is possible to identify and localize binding regions using our methods, despite the fact that they use more limited information and accept less restrictive inputs and are expected to precisely localize the interface. First, we identify calcium ion binding sites to confirm our per-atom classification technique. Second, we explore the impact of training set size in this application. Third, we identify glucose binding sites as glucose is a small and common enough ligand that it can be treated as a single moeity in our method. Finally, we conduct an experiment that considers four ligands to assess the specificity achieved by our approach.

For experiments where we created the training sets, we post-process the training sets found by our algorithm by removing examples deemed homologous with the testing set. For each experiment, we remove members of the training set that were found to have more than 30% sequence identity with the testing set. This post-processing potentially damages the diversity achieved by our sampling process as removing an example effectively removes the representation of the entire cluster. However, it may also improve performance by reducing over-fitting.

#### Per-atom predictor test: calcium ion binding

The performance of the full algorithm is highly dependent of the performance of individual atomic predictors. Therefore, evaluating these predictors forms an important first step toward validating the algorithm as a whole. In this section, the atomic predictor is tested in isolation on calcium ion binding. This choice was not arbitrary: because this type of binding was used as a test subject for FEATURE [45], it serves as a basis of comparison with the predictor described here.

Atom prediction was tested on the 11 proteins that formed FEATURE’s test corpus, listed in Table 2. Training was done using a corpus of proteins which bind to calcium, selected by our algorithm. The training set was post-processed to remove any example that was more then 30% homologous to any member of the testing set. We randomly selected 100 proteins from the post-processed set for training.

**Table 2:** Results from tests of the atomic predictor on finding the binding location of calcium ions

**PDB Code**	**1ANX**	**1AYP**	**1CGV**	**1CLM**	**1OMD**	**3CLN**	**1SAC**	**2SCP**	**3ICB**	**3PAL**	**5CPV**
ROC Area	0.92	0.57	1.00	0.94	0.95	0.97	0.96	0.97	0.96	0.98	0.97

Shown are the 11 proteins used as test cases by Altman et. al [45]. To test these, we first trained a predictor on 100 examples of calcium binding, then evaluated each of the above protein surfaces using this predictor to generate an ROC curve for each test. The number below each PDB code is the area under its respective ROC curve. 1.0 indicates a perfect score, with all true positives found and no false positives. See Figure 1 for illustrations to help interpret these numbers.

Our approach achieved 94% sensitivity at 85% specificity, or 85% sensitivity at 90% specificity. FEATURE reported 91% sensitivity at 100% specificity, however, these numbers may not be directly comparable because we account for the precision of localization differently. Our full results are given in Table 2, which provides the area under the ROC curve for each test case. To help interpret these numbers, three of the results are depicted in Figure 1. Interpretation of these results is provided in the discussion.

#### Testing the impact of training corpus size

In supervised learning tasks, proper training is essential to achieving maximal prediction performance. Having too many examples can result in a predictor that is at best needlessly complicated, and at worst over-fit, thus sacrificing generality. Having too few may also result in poor performance: since we can only identify binding configurations that look like something we have already seen, it is essential that those areas of feature space be adequately sampled.

**Figure 8:**
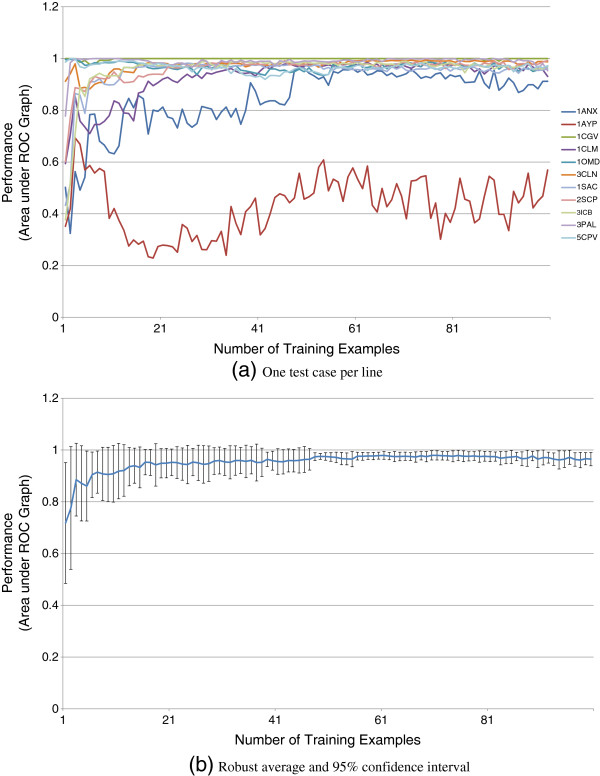
**Shown here is the performance over all test cases of calcium binding (Table**2**) as a function of the size of the training corpus.** Performance is measured by the area under the ROC graph produced by each example. On the top, each test case is shown as a separate line, each with a different color. Note that while with only a few training examples, the correct pocket is found in most tests, a few harder test cases require more training before they can be reliably predicted. On the bottom is the same data, but averaged, with error bars indicating 95% confidence intervals.

To understand this tradeoff, we re-trained the calcium predictor with variably sized subsets of the 100 protein training corpus, with the subsets created by random selection. Results are shown in Figure 8.The results suggest that after enough training examples are seen to properly account for binding variability, a point of diminishing returns is reached quickly, and in fact too many examples may lead to reduced performance (indicative of over-fitting).

#### Small molecule test: glucose

We used our approach to identify glucose binding sites, using the corpus of Nassif, et al. [47]. For this test, glucose was not divided into moeities. We used their training set for this experiment, not the results of our method. The training set consisted of 29 proteins, and their testing set comprised 14. Our methods averaged 86% sensitivity at 93% specificity. This is competitive with their approach, which reported an average of 89% sensitivity with a 93% specificity. Note that our method does not have a separate feature selection step. When Nassif’s does not use feature selection, their performance drops to 76% sensitivity and 84% specificity.

We note that our approach had uneven performance across the testing set. In particular, our approach showed poor sensitivity on two of the examples (1Z8D and 2F2E, at 17% and 26% respectively). As Nassif, et al. do not provide per-example performance, we cannot compare specific cases.

#### Ligand prediction test

To test the entire approach, we selected four ligands (ATP, glucose, DHT and Heme), and created a test set with 10 proteins bound to each.

Here, the complete algorithm is tested, including the moiety prediction steps described above. For this test, four sets of 10 proteins were curated, chosen so that each protein in a set binds to the same ligand, but differently (as much as is possible) from all other proteins in the set. Difference, in this case, was determined by visually assessing the shape and electrostatic distribution on the surface. The PDB codes for proteins in each of these four sets are listed in Table 3. Training corpora were gathered using Algorithm 1 described above. A summary of the training corpora is given in Table 4, which shows how each ligand was broken moieties, the other ligands used to find these moieties, and the number of training examples found for each moiety. These sets were post-processed to cull proteins that with more than 30% sequence identity to any member of the testing set, leading to training sets with between 22 and 318 proteins. 

**Table 3:** Test Ligands and their training sets

**Ligand**	**Test Cases (PDB codes)**
ATP	1A0I 1A82 1ASZ 1E8X 1FMW 1G5T 1GN8 1JWA 1XEX 2BUP
GLC	1AC0 3ACG 1FAE 1GWM 1H5V 1J0K 1JLX 1K9I 2J0Y 2ZX3
DHT	1AFS 1DHT 1I37 1I38 1KDK 2PIO 2PIP 3KLM 3L3X 3L3Z
HEM	1AOQ 1B0B 1CC5 1D0C 1DLY 1EW0 1SOX 1MZ4 2HBG 2Z6F

**Table 4:** Listed are the ligands to be used as test cases, the moieties they contain, the ligands found during training which match each moiety, and the total number of proteins used as training examples after post-process culling

**Ligand**	**Moiety**	**Training Examples**	**#**
ATP	Phosphate Chain	ATP, 5FA, CH1, CSG, CTP, D3T,	36
	(PA O1A O2A O3A PB O1B	DCT, DGT, DTP, GTP, TTP	
	O2B O3B PG O2G O1G O3G)	…	
	Ribose	ATP, 5GP, ACP, ADN, ADP, AMP,	299
	(C1’ C2’ C3’ C4’ O2’ O3’ O4’)	ANP, AP0, APC, ATG, C5P, FAD,	
		GDP, GNP, GTP, NAD, NAP, NDP,	
		RIB, SAH, SAM, SSA, UDP, ADP,	
		…	
	Adenine	ATP, ACO, ACP, ADP, AMP, ANP,	265
	(C2 C4 C5 C6 C8 N1 N3 N6 N7 N9)	ATG, CMP, COA, FAD, NAD, NAP,	
		NDP, SAH, SAM, …	
Glucose	Glucose	GLC	85
HEM	oxygenated end	HEM, DHE, FDE, FDD, HAS, HCO,	316
	(O1A O2A CGA CBA CAA C2A	HDD, HEA, HEB, HEC, HEV, HFM,	
	CMA C3A C1A CHA C4A NA CHB)	HIF, VEA, VER, …	
	non-oxygenated end (1)	HEM, HDD, HDM, HEA, HEB,	318
	(CHB C1B NB CMB C2B C4B	HEC, HFM, HKL, …	
	CHC C3B CAB CBB)		
	non-oxygenated end (2)	HEM, HDD, HDM, HEA, HEB,	318
	(CHD C4C NC CAC C3C C1C	HEC, HFM, HKL, …	
	CHC C2C CBC CMC)		
DHT	(O3 C1-10 C19)	DHT, AE2, AND, C0R, CLR, CPQ,	40
		DXC, FFA, HC2, HCY, STR, TES …	
	(O17 C8 C9 C11-18)	DHT, AE2, AND, ASD, EST, FFA,	22
		TES, WZA …	

**Figure 9:**
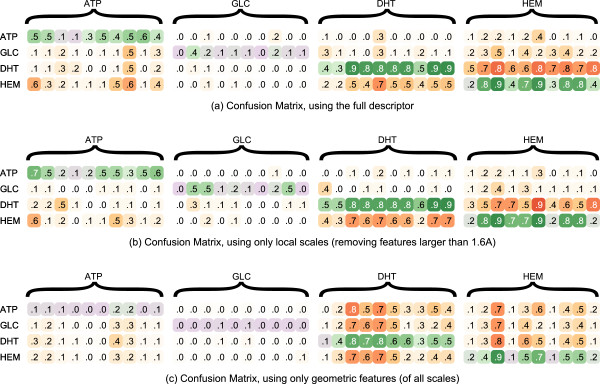
**Shown here are three confusion matrices, thetop (a) tested using the full feature vector description (listed in Table**1**), the middle (b) using only the most local features, and the bottom (c) using only geometric features.** Each row represents the tests for a ligand classifier run on all test cases. Each column represents an individual testing example, grouped by the ligand the protein is known to bind to. The value in the cell is the area underneath the precision/recall curve produced from that test. A higher value indicates a better match. Green cells indicate true positive results: the predictor found the ligand it was trained for. Purple cells indicate false negatives: the ligand failed to find the ligand it was trained for. Red cells indicate false positives: the predictor found the site of a different ligand. See Figure 2 for illustrations to help interpret these numbers.

Figure 9 shows a confusion matrix over all test sets. Here, all 40 test proteins are tested against the four predictors; the value in each cell is the area under the resulting precision/recall (PR) curve. Note that, unlike the above tests, we do not use ROC area. The reason: because the ratio of positive (binding) samples to negative (non-binding) samples varied widely with both the size of the ligand and the size of the protein (and was generally quite low), ROC area did not allow for intuitive comparison between test cases. Precision/recall curves better account for this disparity. Select examples are shown in Figure 2 that convey a sense of how the numbers relate to the detailed results.

Figure 9a shows the results of our method. DHT and HEM are predicted well, although they are often confused. ATP shows more mixed results: though the actual binding site did appear in areas of the highest probabilities on the surface, so did other sites that should not have. The test performed poorly on glucose, although the experiment of the previous section showed good performance on different training and testing sets. These results are considered in the discussion.

#### Descriptor components

To understand the utility of different aspects of the descriptor, two additional experiments used the structure of the previous four ligand test. The first used only the smallest scales in order to show the importance of the “multi-scale” aspect of our descriptor. The second used only the geometric features of our descriptor, to show the importance of considering other types of features. The results as included in Figure 9.

Figure 9b shows the results of only using the smallest scale features. The absence of more global information makes distinguishing DHT and HEM more challenging, but actually improves performance on some cases with small ligands as the larger scale features are simply too big. Improvements suggested by these results will be considered in the discussion.

Figure 9c shows the results using only geometric features, such as curvature and anisotropy. In all cases the results are poor: for DHT and HEM the predictor cannot distinguish between different ligands, and for ATP and GLC the predictor does not identify anything as a probable binding site. This result confirms the need for using features beyond local geometry in surface comparison.

#### Run-times

The time needed to build samples is dominated by three steps: surface tessellation (MSMS), computing electrostatic potential (APBS), and multi-scale shape description. On a 2.8Ghz Core I5 computer with 4gb of RAM, the total time needed to build samples was found to take from 15 seconds for a small protein (1B7V - 70 residues) to 3 minutes for a large protein (1N1H - 1260 residues). The APBS step often dominated, taking well over half the total time. Simpler electrostatics methods may produce adequate results at lower runtime cost, but this was not tested.

Samples, once built, are cached for future use. Each classifier is also serialized and saved to disc, allowing it to be reused. This task, however, is well suited for a distributed approach: surfaces can be independently computed, so each one (or a bundle of them) could be sent out to a separate computer.

Classification performance is highly dependent on both the number of atoms in the training moiety, as well as the size of the protein. Sampling density stays fixed as size is increased; therefore, the number of samples grows linearly with the overall surface area of the geometrical surface of a protein.

Having a large number of surface samples becomes especially problematic during the surface-combination phase, shown in step 2 of Figure 6. This step requires *n*^2^surface function combinations, where n is the number of atoms in the moiety. Each combination requires iterating over every point on one surface and comparing it to a small set of points on another. It is easy to show that the size of this set is a function of both the areal sampling density and the radius of the search. The former is fixed, and the latter is constrained by the physical size of the moiety.

For performance reasons, the surfaces for two atoms that are more than 15Å apart are not combined. Thus the overall time to to combine two surfaces is proportional to the number of samples times the search time. Our implementation uses the Approximate Nearest Neighbor library [66], to accelerate this search process: finding *k* points out of *n* samples with this library takes *O*(*k*·*log*(*n*)). Therefore, combining two surfaces is an *O*(*n*·*log*(*n*)) operation (because *k* is fixed), and combining all surfaces is *O*(*n*^2^·*log*(*n*)).

Surface grouping, therefore, is essential to making the algorithm run fast; with an average 10-fold reduction in the number of samples comes an over 100-fold reduction in run-time. On the system described above, this reduces the time to classify a mid-sized protein from over an hour to less than a minute. In the final algorithm, grouping usually accounts for about one-third of the total classification time; thus, the resulting speedup more than compensates for its cost. Besides the cost of classifying large numbers of samples, the time it takes to classify a given sample using Weka’s LibSVM classifier increases as the number of samples in the training corpus grows.

### Discussion

We feel that the results described in the prior section show the promise of our descriptor-based approach. While the ligand localization experiments may not require the generality of our method, they indicate that the premise of the approach is correct: it is possible to perform function prediction based on functional surface region matching alone; internal structure and global geometry may not be required for matching. The experiments also suggest future improvements to the methods that should improve performance, and make the method viable for a range of applications where generality is useful.

The quantitative metrics we have used to evaluate our approach are problematic. They do not distinguish between the ability to identify that a protein binds a ligand from localizing the interface. They also consider the localization of the interface in a naïve manner that assumes the ligand is stuck in the position frozen in the crystal structure, rather than acknowledging that it more likely moves around within its binding pocket. The quantitative results described in this paper most likely under-state the quality of the results. Even with that caveat, the quantitative results on the ligand localization show encouraging success, but also failures that indicate room for future development.

**Figure 10:**
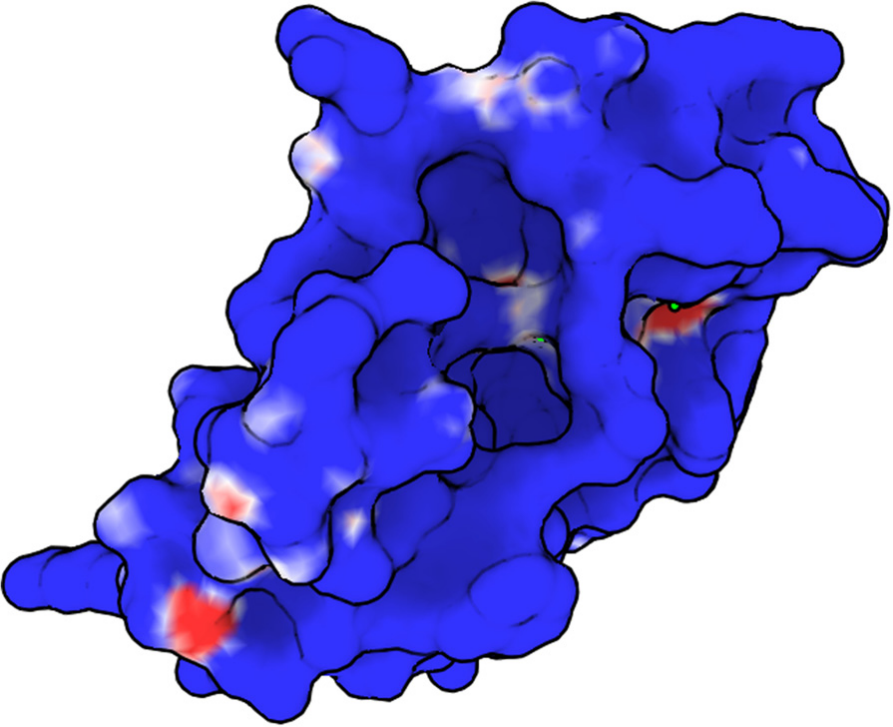
**This image shows the charge (indicated by color, range from dark red (very negative) to dark blue (very positive) of protein 1AYP as computed by APBS.** This charge pattern is quite different than any of the others seen in the training set.

The results of the calcium ion binding experiment are generally successful. In all but one test, our approach is able to localize the binding site well. Interestingly, as many of these sites are on the “outside” of the protein, it is unclear whether pocket-based methods would be as successful. The one failure case in the test (1AYP, shown in the right of Figure 1 and the lowest line in Figure 8a) is a protein unlike any of the 100 others in the training set as it has a positive electrostatic charge over most of its surface (Figure 10). This failure underscores the importance of finding a training set that adequately covers the potential interfaces. Our sampling procedure was designed to find such sets, but relevant examples may have been removed when we randomly reduced the training set size. Also, the sampling process relies on the availability of examples in the PDB and on clustering obtained by BLASTCLUST that considers sequence homology, not functional surface diversity.

The results of the training set size experiment show the tradeoffs in training set size. Enough examples are necessary to cover the different ways the surface may bind, but too many examples can be counterproductive as they create over-fitting. We suspect that better ways of controlling training set size while insuring coverage, for example by using fewer clusters to sample from in the set-selection algorithm, would achieve such behavior.

For the glucose binding experiment, our approach achieves similar results to the method of Nassif, et al. [47]. Curiously, we did not achieve such good results on glucose as part of our larger experiment. The poor performance on glucose seems to stem from its small size. The atomic-predictors are not expected to reliably discriminate between the putative binding of a single atom and its binding with respect to the ligand. Thus, when looking at the probability of a single atom’s binding, there appear to be many false positives. Combining these predictions using the structural information contained in the moiety removes these false positives. But small ligands, like glucose, offer fewer atoms to combine. With larger ligands, the combination approach appears more successful.

The small size of glucose may also contribute to the (relatively) poor performance of the individual atom predictors. The small size of the molecule makes larger scale features inappropriate as they may extend beyond the interface to irrelevant parts of the surface. More generally, our approach relies on being able to adapt to apply the subset of features that are important for specific situations. If the user has some prior knowledge about the features that might be appropriate, that can be used to guide the method. Examples may include limiting the scales for smaller ligands or using experimental knowledge that electrostatics may be particularly important in binding a charged ligand to suggest emphasizing charge features. However, we believe that a better solution would be to employ explicit feature selection, such as used by Nassif et al. [47], that could potentially adapt the feature set based on which ones are likely to be relevant in a given application.

At present, we consider our implementation to be too slow for practical application. Scalability is a concern. However, we believe that these may be addressed by an improved implementation, more intelligent sampling of the surfaces, and applying grouping more widely.

#### Comparison with prior approaches

Functional surface descriptor matching offers the potential to provide a mechanism for function prediction that offers many advantages as described in the Background of this paper. This generality is not necessary for many small molecule binding prediction problems. Existing methods have shown good performance for predicting small molecule binding sites in cases where the pockets are enclosed and the ligands are relatively rigid. We assess our approach on such cases because the prior art provides benchmarks.

Our approach can find regions of the functional surface that can serve as a potential binding site because it has the appropriate physical and chemical properties. This can serve a similar role as a method that determines if an identifiable enclosed pocket is likely to be a binding site for a ligand. Methods specialized to analyzing identified pockets can achieve good performance. While we would not expect our approach to perform as well as more specialized approaches, we look at the fact our approach achieves comparable results as evidence that function prediction is possible based on functional surface region matching alone: that internal structure and global geometry may not be required for matching.

We also stress that our quantitative results are not directly comparable to prior methods as they measure different properties. Our method does not group surface regions into pockets, and our quantification of results includes localization of the binding surface regions. With that caveat, we make some coarse comparisons here to relate our results to those achieved by more specialized, prior methods. While there are many approaches for characterizing and predicting small ligand binding sites, we choose a few representative recent examples here.

Our calcium and glucose binding experiments use the same testing sets as the works of Wei and Altman [45] and Nassif et al. [47] respectively. In both cases, the authors show that they can achieve excellent performance with specialized methods (the former requires spherical shells, while the latter requires small enough targets that a single descriptor can capture the entire binding pocket). Our approach is successful on almost of all their examples, though with a much more general approach.

Kahraman and Thorton [29] describe a method for assessing the similarity of binding pockets based on a comparison of the spherical-harmonics decomposition of each pocket. The authors use this descriptor to test a diverse set of binding pockets, including those for ATP, Androgen (similar to DHT), HEM and glucose.

Their primary conclusion is that the shape of the binding pockets for a given ligand varies more than the shape of the ligand itself. This means that the shape variation of a pocket is greater than can be accounted for by ligand flexibility, and thus complementarity is neither necessary nor sufficient for predicting ligand binding.

To support this conclusion, they show that, using a spherical-harmonic description of the pocket, the shape of ATP, AMP and steroid pockets are easily confused with one another. Glucose pockets, on the other hand, are not often confused with anything but phosphate, thus indicating that size does matter for pocket comparison. In contrast, our method does not use global measurements or descriptors of the pocket, making it applicable in applications where the target interface is not an enclosed region. However, in cases where global descriptions of shape are available and adequate, our approach does not perform as well. For example, our method confuses DHT and HEM, though their method suggests that the shape of HEM pockets is unlike that of any other pocket.

The method recently introduced by Chikhi and Kihara [67] is designed to allow for extremely quick lookup of protein structures, using a query structure as a template. Their method requires a few minutes per protein of preparation time to build the surface, identify pockets and compute 3D Zernike moments. But after preprocessing, they claim that their database can return back the results of a query against hundreds of proteins in a few seconds. So while the preprocessing cost for a single protein in their method is comparable to ours, theirs is several orders of magnitude faster for search.

The matching process of Chikhi and Kihara [67] combines the Zernike moments with other global measurements of pocket shape to achieve excellent performance at identifying pockets for ligands when these sites are amenable to such global description. They evaluate the percentage of queries in which the searched-for pocket is returned back as the top result or is in the top 3 results. They test on several datasets, including the Kahraman set [29], which contains three of the same ligands as ours: ATP, GLC and HEM. For these, on average, their method (using both pocket shape/size and electrostatic potential) finds the correct pocket on the first try 57%, 40% and 50% of the time, respectively. When expanding to the top three picks, their method improves to 92%, 100% and 86%.

Unfortunately, the statistics they provide for their results are not easily comparable to those presented here. Because our method is not pocket-centric, it is not possible to account for results in the same way. For example, in Figure 2 f and h, our approach correctly identifies the pocket, but get low scores because they fail to confidently and precisely localize the binding interface within this pocket. To make a crude comparison with pocket centric metrics, we can consider their “top-1” cases to be highly confident success, and the “top-3” cases to be confident success at identifying the ligand pocket. Similarly we can say our method successfully finds a pocket when there are true positives found at the given confidence level. For a high confidence (85%), the identified region contains the target ligand 82%, 71% and 97% of the time (for ATP, GLC, and HEM respectively) For an extremely high confidence value (95%), the identified region contains the ligand 60%, 55% and 83% of the time.

Again, we would not expect our method to provide the performance of more specialized methods. However, our method’s performance on problems for which benchmarks are available suggest that it is a viable approach. With the performance improvements suggested above, including feature selection and improved training set generation, we expect that the local functional descriptor approach will be valuable in applications where the specialized approaches are not appropriate. We are particularly interested in exploring the application of our approach to interfaces between proteins, or between proteins and other macromolecules. In such applications, the interfaces are not enclosed pockets, so pocket specialized approaches cannot be readily applied.

## Conclusions

In this paper, we have introduced a new descriptor for protein functional surfaces, and demonstrated it in identifying ligand binding interfaces. The descriptor captures the local geometric, chemical, and physical properties of the functional surface, independently of the internal configurations of the atoms that create these fields. Our initial applications of these descriptors show that they can serve in binding prediction tasks, despite only capturing functional surface properties. In the future, we expect to develop more sophisticated algorithms that will employ these descriptors to provide better performance on a range of recognition prediction applications.

## Endnote

^a^By shape, we take the standard meaning of the space from which other molecules are excluded from the protein. Abstractly, the spatial arrangements of other physical properties are also “shape,” however, we reserve the term for its common usage.

## Competing interests

The authors declare that they have no competing interests.

## Authors’ contributions

All authors contributed to the development of the approach. GC implemented the system and ran the experiments, and wrote an initial description as part of his dissertation. MG re-wrote the content into paper form. All authors read and approved the final manuscript.
